# Thermal Peak Management Using Organic Phase Change Materials for Latent Heat Storage in Electronic Applications

**DOI:** 10.3390/ma11010031

**Published:** 2017-12-26

**Authors:** Jacob Maxa, Andrej Novikov, Mathias Nowottnick

**Affiliations:** Institute of Electronic Appliances and Circuits, University of Rostock, Albert-Einstein-Str. 2, 18059 Rostock, Germany; Andrej.Novikov@uni-rostock.de (A.N.); mathias.nowottnick@uni-rostock.de (M.N.)

**Keywords:** thermal management, phase change materials, latent heat, reliability

## Abstract

Modern high power electronics devices consists of a large amount of integrated circuits for switching and supply applications. Beside the benefits, the technology exhibits the problem of an ever increasing power density. Nowadays, heat sinks that are directly mounted on a device, are used to reduce the on-chip temperature and dissipate the thermal energy to the environment. This paper presents a concept of a composite coating for electronic components on printed circuit boards or electronic assemblies that is able to buffer a certain amount of thermal energy, dissipated from a device. The idea is to suppress temperature peaks in electronic components during load peaks or electronic shorts, which otherwise could damage or destroy the device, by using a phase change material to buffer the thermal energy. The phase change material coating could be directly applied on the chip package or the PCB using different mechanical retaining jigs.

## 1. Introduction

The trend in electronic assemblies to accommodate higher power conversion in smaller packages leads to an imbalance in thermal management. Despite the continuous shrinking of electronic parts on a printed circuit board (PCB), the thermal loss of those parts remains the same or is going to increase [1]. This leads to smaller heat transfer interfaces between the actual electronics and the surrounding environment. This fact shows the urgent demand for an effective thermal management and cooling system on electronic assemblies.

A similar problem occurs when it comes to power electronics, such as power transistors, rectifiers, Insulated Gate Bipolar Transistors (IGBTs) or motor control circuits. These components are manufactured with a factory-given power dissipation exceeding 100 W on less than 1 cm${}^{2}$. This energy in form of heat has to be diverted from the device to avoid de-rating or damage.

The common approach to handle this topic is to add cooling elements, such as heat pipes, fans, heat spreaders or a combination of them, to improve the thermal heat flow from the device to the environment. Those large components, mostly made of metals or ceramics, require a significant amount of space within the assembly and in the device housing. Additionally, it raises the total weight of the whole system as well as the maintenance expenses and production costs.

Every additional part of a heat sink on a PCB increases the footprint for its placement and fixture. Moreover, thermally conductive pads or compounds are required to fill any air gaps between the heat sink and the component. This leads to an increased effort in design, manufacturing, assembly, and costs.

In order to overcome these challenges, there is a demand for a more effective way to tackle the thermal management problem of today’s electronic assemblies. This paper will introduce a novel approach to handle the thermal challenges by using the latent heat storage in a phase change material (PCM) with a direct on-board applied coating. This will flatten the thermal peaks in electronic components with no need for any additional dedicated PCM package or filled heat sink. This paper is organized as follows: Section 2 will provide the basics behind latent heat storage, while Section 3 introduces the new approach of the cooling concept. The screened materials will be presented in Section 4, their fixations in Section 5. The measurement system is introduced in Section 6 and gets evaluated in Section 7.

## 2. Fundamentals

### 2.1. Phase Change Materials

The group of phase change materials consists of elements or compounds that undergo a phase change when they reach a certain temperature. They can be classified in four categories: solid-solid transition, solid-liquid transition, solid-gas transition and liquid-gas transition. Depending on the material, each phase transition occurs at a distinct characteristic temperature. Water for example has three transitions:

- solid-liqid at 0 ${}^{{}^{\circ}}C$ (1 bar): ice melting to water
- liquid-gas at 100 ${}^{{}^{\circ}}C$ (1 bar): water boiling to vapour
- solid-gas at 0.01 ${}^{{}^{\circ}}C$ ( 0.006 bar) ice sublimating to vapour

The phase transitions can be traversed in both directions, with energy either released to or absorbed from the environment. The energy can be thermodynamically represented by a change in temperature, pressure or volume. In most cases the temperature variance has the major impact on a phase change [2].

**Figure 1:**
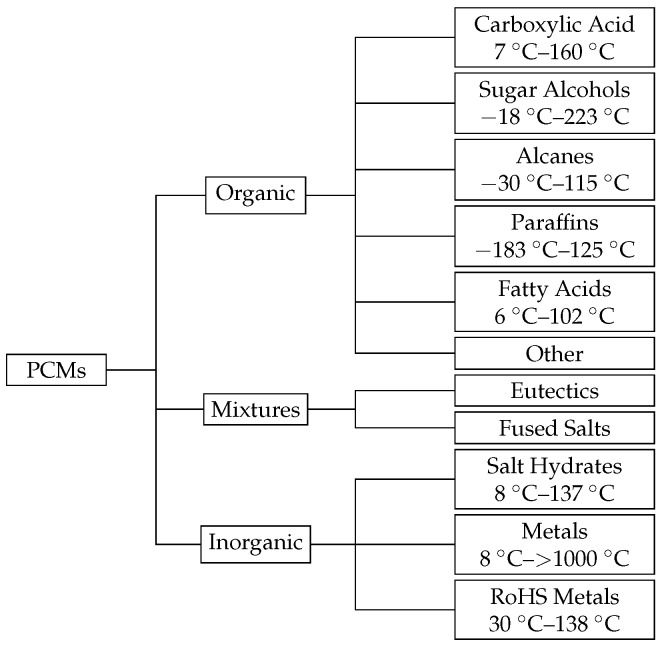
Overview of solid-liquid PCMs with their typical melting points at 1 bar [4,8].

A wide range of PCM materials are commercially available and can be classified into multiple classes (Figure 1).

One of the current research topics is to find and characterize new materials that can be used as a suitable PCM for a specific application [3,4,5,6,7]. Regarding a special application, many constraints must be satisfied by a single PCM. Especially for the targeted application, there is the demand for a low volume and pressure change and non-volatility of the material. Therefore, only PCMs that possess a solid-liquid phase transition are considered.

### 2.2. Latent Heat Storage

Heat can be stored by a material as either sensible heat or latent heat. In the case of sensible heat $Q_{s}$, no phase transition is involved, the amount of stored thermal energy *Q* is given by the specific heat $c_{p}$ of the material as Equation (1) describes.

(1) $$\begin{array}{c} \Delta Q_{s}=C\;\cdot \;\Delta T=m\int_{T_{low}}^{T_{high}}c_{p}\;dT=m\;\cdot \;c_{p}\;\cdot \;\Delta T \end{array}$$

**Figure 2:**
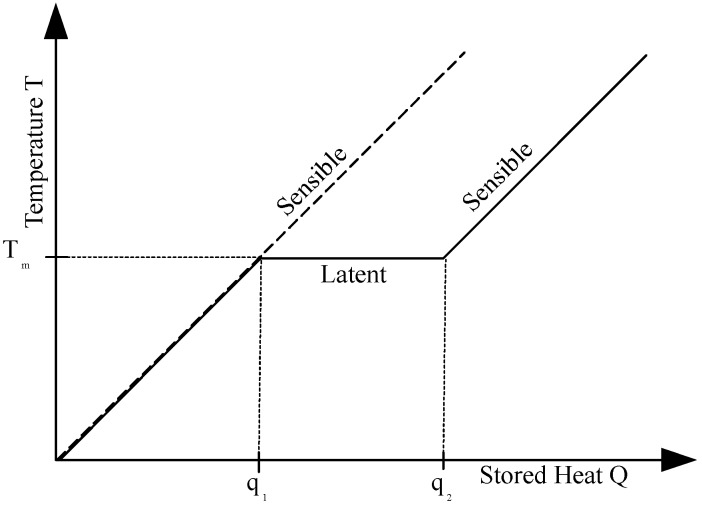
Sensible and latent heat storage for a phase change.

In contrast, in case of a latent heat storage, the energy is required to release the molecular bonds of the material in the phase transition. The temperature remains nearly constant at the melting temperature $T_{m}$, until the whole mass has completed its phase change. This latent heat storage $Q_{l}$ (Equation (4)) absorbs the heat while keeping the temperature of the material constant as Figure 2 demonstrates. The symbol $a_{m}$ denotes the fraction of melt, with $a_{m}=0$ corresponding to fully solid material and $a_{m}=1$ corresponding to fully molten material.

(2) $$\begin{array}{cc} \Delta Q & =\Delta Q_{l}+\Delta Q_{s} \end{array}$$

(3) $$\begin{array}{cc} \Delta Q & =\underset{\mathrm{latent}\;\Delta Q_{l}}{\underset{︸}{m\;\cdot \;a_{m}\Delta h_{fus}}}+\underset{\mathrm{sensible}\;\Delta Q_{s}}{\underset{︸}{\int_{T_{s}}^{T_{m}}m\;\cdot \;c_{p,s}\;dT+\int_{T_{m}}^{T_{l}}m\;\cdot \;c_{p,l}\;dT}} \end{array}$$

(4) $$\begin{array}{c} =m[a_{m}\Delta h_{fus}+c_{p,s}\left(T_{m}-T_{s}\right)+c_{p,l}\left(T_{l}-T_{m}\right)]\; \end{array}$$

The amount of stored heat is proportional to the enthalpy of fusion $\Delta Q_{l}=q_{2}-q_{1}∼\Delta h_{\mathrm{fus}}$ for isobaric transitions.

### 2.3. Previous Research and Research Objectives

The concept of employing PCMs for cooling applications in electronics has been established in research over the recent years [9,10,11,12,13]. These previous ideas aim at encapsulating PCMs in a container or cavity that is directly thermally coupled to the electronic component. Advantages of this concept are the large amount of PCM that can be used and the fixture of the liquid material. Green et al. [13] encapsulate a small amount of PCM in a wafer level chip cavity to extend the duty cycle of switching applications.

Other concepts increase the thermal conductivity by adding metallic structures, such as foams, honeycomb plates, fabrics or fins to improve the melting speed gradient [12,14,15]. Furthermore, graphene and similar carbon derivatives are investigated and used as heat conducting structures in the form of foams or agglomerates [16,17,18]. Even ceramic materials were investigated for this purpose [15,19,20].

Mostly driven by solar energy storage applications, PCMs, and in particular the sugar alcohols as a solid-liquid PCM, get into focus of researchers worldwide. In consequence, more and more materials get proposed as a PCM and become available on the market. Especially the eutectic mixtures - material mixtures with a certain composition out of two or more materials with a special temperature point, where each material melts to the liquid phase - are in focus of the researchers [21]. Their advantage is the possibility to choose a composition with a user selected melting point.

This research will focus on a direct applicability of a coating for existing electronic components and assemblies in a macroscopic dimension. The coating composite material should be applied directly during the PCB manufacturing process without the need for a dedicated and complex additional production step. As few as possible design guidelines, constraints or technology should be required.

## 3. Cooling Concept

**Figure 3:**
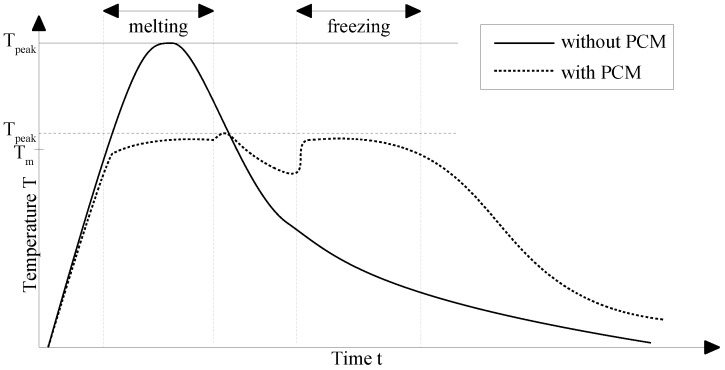
Generic theoretical temperature peak limiting example chart with and without an applied PCM.

Figure 3 displays a generic example of the introduced concept. By absorbing the thermal energy of a device during a phase transition, the maximum temperature $T_{\mathrm{peak}}$ is limited by the melting temperature $T_{m}$ of the PCM.

### 3.1. Requirements

As mentioned in the introduction, every special application requires some constraints regarding the final product as summarized in Table 1.

Furthermore, the material must be inflammable, non-conductive, non-toxic, non-corrosive, must have a low hygroscopic behaviour and be commercially available. When screening possible PCMs available, the group of sugar alcohols stands out to fulfil the requirements.

Another condition is the ability of the material to remain at its position after their application on a final product during the whole lifetime. Since these materials have a very low viscosity in their liquid state, an additional fixture will be required for this purpose.

Comparing the thermal conductivity from the requirements with the values of the sugar alcohol from the literature [22] ($\lambda \approx 0.73Wm^{-1}K^{-1}$, decreasing with higher temperature), it is conspicuous that this property has to be improved by additional filler materials with a better thermal conductivity.

### 3.2. General Description

Compared to previous publications, this paper describes a direct application of a solid-liquid PCM on the electronic component or assembly. Thus, the electronic part or assembly should directly be covered with the PCM to absorb the thermal energy during its normal operation, without the demand of a solid encapsulation container.

To achieve the requirements from Section 3.1 with the group of the sugar alcohols, some further investigations are required. By adding different materials, some physical properties of the sugar alcohols can be modified to fit these requirements. Those could consist of different materials like copper (Cu), carbon nanotubes (C) or aluminium oxide (${\mathrm{Al}}_{2}O_{3}$). Each material interacts differently with the PCM with the advantage to improve more than only a single property. The details of the influence of different materials are presented in the next section. The combination of the actual PCM with the additives result in a new material called Phase Change Composite (PCC).

In addition to optimizing the parameters, the PCM/PCC must be fixed at its position during the liquid phase. Some approaches to accomplish this objective will be presented in Section 5.

## 4. Material Evaluation

### 4.1. Sugar Alcohols

Sugar alcohols as solid-liquid PCMs exist in a crystalline form at room temperature. To screen the different sugar alcohols from Table 2, each of them were melted twice: at first after filling approximately 1.5 g in small test tubes of borosilicate glass and heat them up to 30 K above their melting temperature within a heated glycerol bath. The second measurement was processed in groups with nearly the same melting range within an oven. In an oven, it is advantageous to have a homogeneously distributed temperature within the ventilated oven chamber and a constant and equal heating rate for each sample tube.

Sorbitol, Pentaerythritol and Dulcitol did not fit the required enthalpy (compare Table 1) but were investigated for additional tasks. With the formation of eutectic mixtures (see next paragraph) these materials can be combined with other sugar alcohols to create a compound that will satisfy this requirements.

#### 4.1.1. Simulation

As mentioned earlier, sugar alcohols form eutectic mixtures with a defined and predictable melting temperature in dependence of the molar fraction of the components. The base for the calculations provides the modified UNIFAC (Universal Quasichemical Functional Group Activity Coefficients) activity model (see [23]), which describe a physical behaviour correlation between similar chemical molecular groups. This model gives the activity coefficients $\gamma =f(\chi_{a},\chi_{b})$ for each material combination of $\chi_{a}$ and $\chi_{b}$ by an observation of functional groups of particular materials.

(5) $$\begin{array}{cc} \Psi & =\sum_{i=a,b}^{}\chi_{i}\;\cdot \;\gamma_{i}=1\; \end{array}$$

(6) $$\begin{array}{cc} & =\sum_{i=a,b}^{}\exp\left[\left(\frac{\Delta h_{fus,mi}}{R\;\cdot \;T_{mi}}\right)\left(\frac{T-T_{mi}}{T}\right)\right]-\frac{\Delta c_{p,i}\left(T_{\mathrm{mi}}-T\right)}{R\;\cdot \;T_{mi}}+\frac{\Delta c_{p,i}}{R}\ln\frac{T}{T_{mi}} \end{array}$$

(7) $$\begin{array}{cc} \Psi & \overset{\Delta c_{p,i}\to 0}{=}\sum_{i=a,b}^{}\exp\left[\left(\frac{\Delta h_{\mathrm{fus},\mathrm{mi}}}{R\;\cdot \;T_{mi}}\right)\left(\frac{T-T_{mi}}{T}\right)\right] \end{array}$$

(8) $$\begin{array}{cc} \Psi_{eut} & \to \Psi_{a}=\Psi_{b}=min\left(\Psi \right) \end{array}$$

The composition Ψ describes the components molar fraction $\chi_{i}$ with the activity level $\gamma_{i}$. Since the change of the specific heat during the phase transition is negligible ($\Delta c_{p,i}\to 0$), the terms of Equation (6) simplify to Equation (7). The eutectic point results from the intersection of the liquidus line from component *a* and *b*.

(9) $$\begin{array}{c} \Delta h_{fus,mix}=\sum_{i=a,b}\chi_{i}\;\cdot \;\Delta h_{fus,i} \end{array}$$

**Figure 4:**
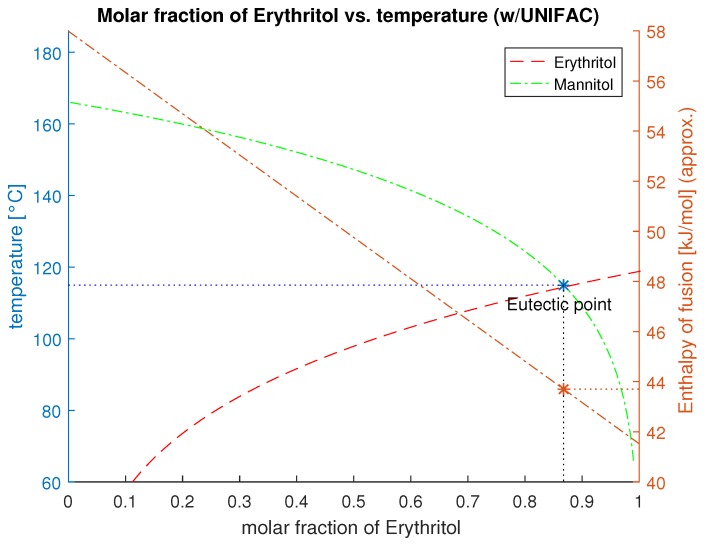
Phase diagram and liquidus line of the mixture Erythritol and D-Mannitol with their eutectic melting temperature and resulting enthalpy of fusion.

The resulting enthalpy can be calculated by Equation (9). Plotting these values results in a phase diagram as in Figure 4.

A MATLAB script was developed to observe the sugar alcohols of interest, comparing different mixtures with respect to the requirements mentioned in Section 3.1. The results of this script were presented in Table A1 in the Appendix A of this paper.

#### 4.1.2. Measurement

Melting and freezing curves were measured for each pure material. Every sample was put in a convection oven and heated up at constant rate of about 13 K $\mathrm{mi}n^{-1}$.

**Figure 5:**
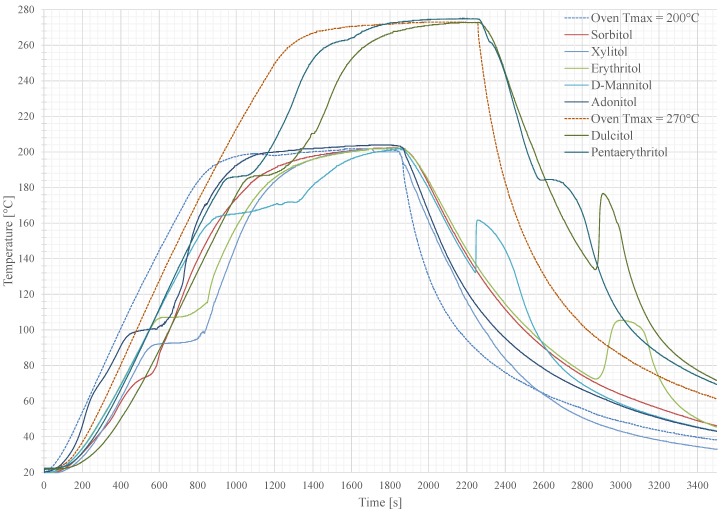
Temperature curves over time of the screened sugar alcohols with heat up and cool down phase. Each plateau in the heating part describes the melting process, where the temperature stays constant. The second plateau is reported as the material reached the ambient oven temperature. In the cooling state, the additional heating is caused by the recrystallisation of the PCM where the stored heat is released.

To avoid a vaporisation of the sample materials, two groups were classified with different peak temperatures of 200 ${}^{{}^{\circ}}C$ (group A) and 270 ${}^{{}^{\circ}}C$ (group B). The results are shown in Figure 5. Each PCM keeps its temperature at its melting point until the whole material is completely molten. Afterwards, the sensible temperature rise continues. The second plateau is reached, when the sample hit the maximum ambient oven temperature. The area between the oven curve and the material curve can be seen as a measure to compare the materials for its applicability as a temperature peak buffer. After the heating of the oven is turned off, the oven door was opened to let the samples immediately cool down to room temperature (approximately 20 ${}^{{}^{\circ}}C$). The visible subcooling, described by the temperature difference between the melting point and the start of the crystallisation, is different for each sample. Dulcitol, Pentaerythritol, D-Mannitol and Erythritol show a spontaneous and fast crystallisation while the other samples have a low crystallisation dynamic and require more than an hour to reach a complete solidification.

### 4.2. Additives

Within literature there is hardly any information available regarding the thermal conductivity of the selected sugar alcohols, except for Erythritol with $\lambda \approx 0.73Wm^{-1}K^{-1}$. Due to the chemical and physical structural resemblance of all sugar alcohols, all of them are assumed to have a value in this range.

The heat in the PCM only transfers through conduction within the crystallized material until it starts melting. The initial melting front initiates on the interface between the heat source and the PCM, and continues radially through the PCM volume with a velocity that is limited by the thermal conductivity. Thus, the conduction of heat within the material is insufficient for a direct application without any enhancements by additives with a significant higher heat conductivity. Agglomeration and mixing of those additives with the PCM is an important condition.

An optimal additive creates a continuous matrix, where the main structure consists of a highly thermally conductive material that expands the thermal interface between the heating source and the PCM. As a consequence the interface between a heat source and the additive requires a tight thermal connection. Care must be taken when electrically conductive additives are used. In this case, an insulation layer must be added to the electrical assembly or component to prevent short circuits.

#### 4.2.1. Powders

Powders of metals or ceramics provide a good miscibility with the PCM and a good thermal conductivity. However, in contrast, they tend to sediment because of their higher physical density. Additionally, they require a high packing density in the composition, to create a thermally conductive matrix, resulting in a reduced fraction of PCM in the composite. Seven additives in powder form were mixed with Xylitol as an example for a PCM. In addition to metals and ceramics, even other PCMs with an alternative melting point were evaluated as a nucleus for crystallisation.

As expected, the metals increase the crystallisation rate. Exact measurements of the thermal conductivity and crystal growth rates of the mixtures are outstanding and will be reviewed in future research.

As already seen in first investigations (compare Table 3), the metal powders with particle size from 5 to 200 μm indicate a good miscibility with the PCM. Carbon Nano Tubes (CNT) in contrast tend segregate at the bottom of the test tube or float on top of the liquid PCM. No full encapsulation of the CNT within the PCM was noticeable [27]. To achieve a homogeneous distribution of these particles within a PCM, special surface treatments of them are required, such as plasma coating.

The observed effect of metal powder allows a conclusion to the nucleation of the sugar alcohols regarding a minimal nucleus size required for a beginning crystallisation. These materials exhibit a high surface energy that supports the heterogeneous nucleation of the surrounding PCM [28].

#### 4.2.2. Liquids

Also kindred structured materials were proposed to improve the crystallisation rate of sugar alcohols. Sepplälä et al. discovered an acceleration of the crystallisation rate by the factor 33 with 10% Methanol as an additive for Xylitol [29]. Due to chemical similarities among all sugar alcohols, it is probably possible to expand this reaction to other materials of this group. The combination of different sugar alcohols in their liquid phase could improve the thermal endurance and cycle stability as shown in [30].

## 5. PCM Enclosures

An enclosure serves a triple purpose. First, it encapsulates the PCM in the solid and liquid state and prevents it from flowing away. Second, it creates a thermal embedding or matrix to improve the thermal conductivity. The third task is to expand the surface for additional nuclei for initial crystallisation. Furthermore, an enclosure must protect the PCM from environmental influences, such as moisture or ultraviolet light. The following paragraphs describe different ideas so solve these issues.

### 5.1. Resin Matrix

It is possible to create a composite with a PCM and an epoxy or acrylic resin matrix. The PCM must be ground in the solid phase and stirred with the resin in their liquid phase. After an application on the electronic component or PCB the material is getting cured and forms a solid composite.

**Figure 6:**
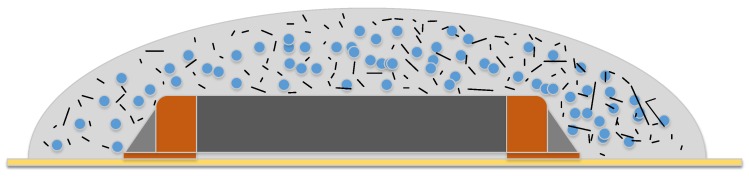
Example of a resin fixation matrix (light grey) with additives (black strings) and the PCM (blue) on top of a chip resistor (orange/dark grey), soldered on a PCB (yellow).

Previous research [27] examines multiple combinations of Erythritol and an electronic covering coating with best results with a volume content of 55 wt. % to 60 wt. % of Erythritol to coating mixture. An increase of the Erythritol content leads to high viscosity and didn’t allow the application of the composite on the target component. Figure 6 demonstrates this concept.

### 5.2. Dam and Fill

**Figure 7:**
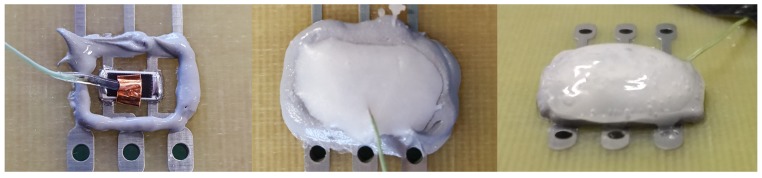
Dam and Fill example on a 2512 chip resistor. **Left**—mounted resistor with single dam structure, **Middle**—added PCM, **Right**—with applied PCM and Glob Top capping.

The Dam and Fill fixation consists of three components: the dam made of a resin matrix, that surrounds the component on the PCB on a vertical plane, the PCM that fills the cavity within the dam and a Glob Top that encloses the PCM and prevents a drain of the molten PCM. The task of the dam is to fix the PCM in the solid and liquid state at the position. The Glob Top capping layers must do the same but additionally adjust the difference in volume of the PCM during melting and crystallization. There are some constraints on the dam and Glob Top material. These must be temperature stable over the full operating range of the electronic component. Furthermore, it must be resilient (elastic), but not brittle to stress and relax with the volume change. Figure 7 demonstrates the application steps on an SMD 2512 chip resistor in lab scale. Each step was carried out manually. The PCM to fixation ratio is better compared to the resin fixation. Here the PCM made more than 80 wt. % of the compound, the cooling efficiency is higher.

### 5.3. Cooling Moulding

A third approach is to completely encapsulate the electronic component or assembly within a container, filled with a PCM/PCC. Since the material must be electrically insulating and non-corrosive, this fixation is able to cover a complete electronic assembly. The container must consists of different materials that are capable to transport the heat from the assembly away. This fixation could handle the largest volume of the PCM compared with the previously described ones and could buffer the largest amount of latent heat.

**Figure 8:**
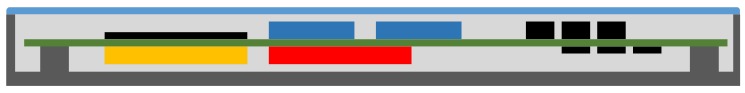
Cooling Moulding fixation for full volume PCM applications. The green PCB in the middle contains double-sided components. The light grey symbolizes the PCM while the blue Glob Top represents the cover of the container.

As Figure 8 illustrates the whole electronic assembly is immersed in the PCM and covered with a flexible panel that regulates the expansions and shrinking of the PCM during its operation cycle.

## 6. Measurement Circuit

**Figure 9:**
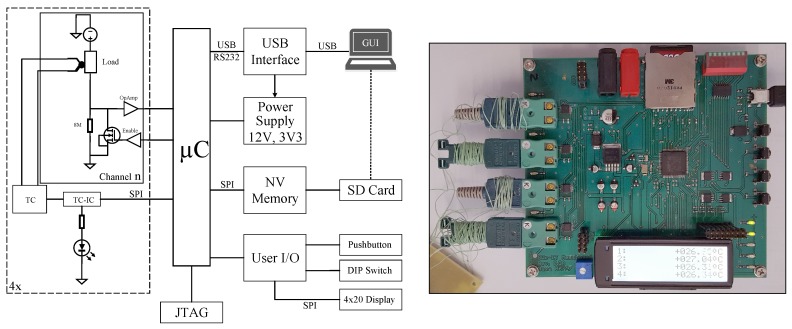
**Left:** Measurement circuit for test components with load control and temperature measurement. **Right:** Fully assembled measurement PCB.

A testing circuit with four independent channels was developed to use for multiple purposes. The primary objective of this platform is, to investigate the thermal stress of various component packages with an applied PCM composite coating. The electronic parts are heated by creating a controlled electrical short-circuit within the device. Simultaneously, the component’s temperature is measured using a K-type thermocouple per channel as Figure 9 demonstrates. The load component could be a simple resistor or a field effect transistor (FET) that is controlled by a pulse width modulation (PWM) driven enable signal.

The measurement board can be connected and controlled by a PC or a configuration file, stored on an SD memory card. Measured data could also be stored on this SD card. By using this setup it is possible to create different load and cycle profiles for each of the four available channels. To create a configuration file, stored on the SD card, a graphical user interface (GUI) , written in Python, can be used. Furthermore, it can control the hardware directly trough the USB connection.

This measurement system is used to carry out long term cycle tests of different PCM composites and their application methods on the electronic component or PCB. It can be used independently of a computer and only requires a power supply. Each channel could create a thermal loss up to 48 W.

## 7. Measurements and Recommendations

**Figure 10:**
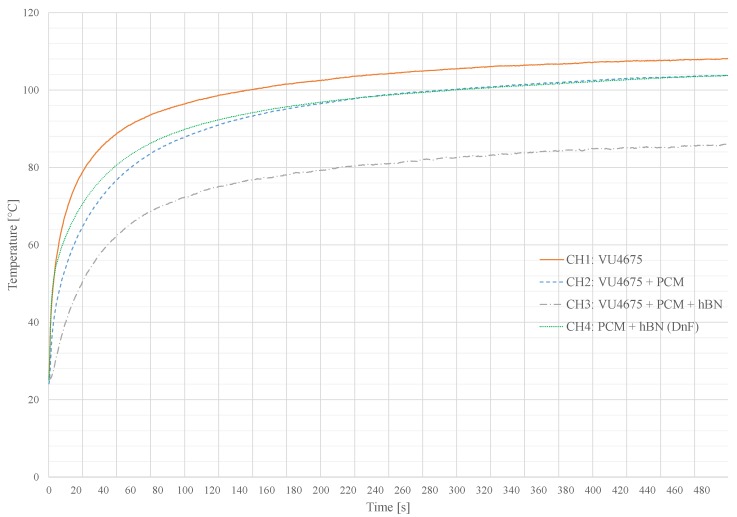
Temperature-time plot for different PCM enclosures on the measurement system. Each channel is heated with 1.4W. A 2512 chip resistor is used as a heater. The temperature was measured directly on the component package with a K-type thermocouple.

Figure 10 shows four different measurements, recorded with the system presented in Section 6. Each channel is equipped with a 100 Ω resistor in a 2512 ceramic package. Channels one to three were coated with a matrix made of a silicone-based casting compound VU 4675 by Lackwerke Peters. It has a thermal conductivity of $\lambda =1.2Wm^{-1}K^{-1}$ due to the addition of alumina oxide particles. The capping layer and dam structures from channel four were also made of this material. The thermal conductivity of this compound can further be improved by adding up to 10 wt. % hBN.

As listed in Table 4, channel one is the reference value without any PCM or additive. Thus, this channel reaches the highest temperature of 108 ${}^{{}^{\circ}}C$. Both, channel two and four reach an equilibrium with a maximum temperature of 103 ${}^{{}^{\circ}}C$. The Dam and Fill application in channel four has a faster heating during the heating period, compared to the similar composite compound in channel three, based on the improved thermal conduction of the material mixture of PCM and Hex-Boron Nitride (hBN).

The most efficient cooling is observable in channel three, consisting of a composite of casting compound, PCM and hBN for a thermal enhancement. Heat generated by the component is spread and emitted to the ambient before the PCM starts to absorb the thermal energy by melting. The maximum temperature after 500 s heating period was 86 ${}^{{}^{\circ}}C$. The silicone-based Glob Top capping applied on this channel could withstand the thermal volume change for at least 25 cycles ($t_{heating}$ = 500 s, $t_{cool}$ = 2500 s). Climate and long term tests are pending.

Further series of measurement show similar results. As a component heats up quickly, the Dam and Fill enclosure is advisable, concerning the fast thermal conductance of the PCC with a quick melting reaction of the PCM. On a slower but permanent heating, the matrix application with thermal enhancements and PCM components is preferable. To select an optimal enclosure for a target application, the decision hardly depends on the primary objective. When using a large component package and sufficient free area around the component is available, the Dam and Fill method is preferred, because the larger amount of applicable PCM and the huge thermal interface.

If the PCB layout is fixed and the area to be coated is unsuitable to build a dam structure, then the matrix approach has the best effort. Also, when small sized packages such as chip resistors or capacitors are used, a small amount of matrix encapsulated PCM could improve the thermal behaviour and reduce the peak temperature.

## 8. Conclusions and Future Work

The concept of a thermal buffer coating for electronic assemblies and components was presented in this paper. Sugar alcohols were selected as a PCM that can accomplish the requirements on a latent heat buffer material for electronic applications. The presented encapsulations were examined in initial investigations for their feasibility. A selection from the presented additives, in particular the Hexagonal Boron Nitride (hBN), shows promising properties to improve the PCM and form a suitable PCC. Furthermore, an exact screening of the additives is required, primarily to improve thermal conduction of the PCC. Characterising the volume change, the cycle-ability and the enthalpy alteration after multiple cycles of melting and solidification of the used PCMs during their operation is mandatory to ensure the long term stability and reliability. Tests are currently running at the date of this publication and will be a subject in future works. The presented measurement setup is used for these investigations. Multiple device packages should be characterized and tested.

First results show a realizable solution for the objective to find a coating for PCB applications as a temperature limiting material, relying on sensible and latent heat storage. The peak temperature could be limited by 22 ${}^{{}^{\circ}}C$ even without an optimal PCM selection and thermal improvement. 

## Figures and Tables

**Table 1:** Requirements.

Property	Value	Unit
Enthalpy $\Delta h_{\mathrm{fus}}$	>200	J g${}^{-1}$
Melting Point $T_{m}$	80–100, 140–150	${}^{{}^{\circ}}$C
Reuseability *n*	>5000	cycles
Thermal Stability $T_{destr}$	$T_{m}$ + 50	${}^{{}^{\circ}}$C
Thermal Conductivity λ	>1.5	W m${}^{-1}\;$K${}^{-1}$
Electrical Resistance *R*	>10${}^{7}$	$\Omega \;{\mathrm{cm}}^{-1}$
Hold Time $t_{h}$	$30\leq t_{h}\leq 300$	s

**Table 2:** Screened sugar alcohols.

Name	Formula	Melting Range	Enthalpy	Density
Unit	-	°C	J g^−1^	g cm^−3^
Sorbitol	$C_{6}H_{14}O_{6}$	68–74	165.75	1.49
Xylitol	$C_{5}H_{12}O_{5}$	88–93	210.25	1.52
Adonitol	$C_{5}H_{12}O_{5}$	95–103	239.37	1.50
Erythritol	$C_{4}H_{10}O_{4}$	106–118	354.7	1.45
D-Mannitol	$C_{6}H_{14}O_{6}$	162–172	326.8	1.52
Pentaerythritol	$C_{5}H_{12}O_{4}$	184–189	36.29	1.40
Dulcitol	$C_{6}H_{14}O_{6}$	185–189	119.28	1.60

**Table 3:** Influence of screened additive powders on Xylitol as an example PCM to increase the thermal conductivity and the crystallization rate. Legend: +: better/improved; −: worse/deteriorated; o: no change.

Additive	λ (W m${}^{-1}$ K${}^{-1}$)	Miscibility	Crystallisation	Source
Copper	401	+	+	[24]
MgCl + 6H${}_{2}$O	0.704	−	o	[8]
Zinc	110	+	+	[24]
MWCNT	>2000	−	−	[25,26]
Nickel	85	+	+	[24]
Palmitic Acid	0.162	−	−	[8]
${\mathrm{Al}}_{2}O_{3}$ Ceramic	40	+	+	[26]
Hex-Boron Nitride	40	+	+	[26]

${}^{a}$ Only in longitudinal axis; ${}^{b}$ Only in planar axis.

**Table 4:** Experimental parameters for enclosure comparison.

Channel	Application	*T*${}_{0}$	PCM	Additive	*T*${}_{\max}$	Δ*T*
1	Matrix	22${}^{{}^{\circ}}C$	-	-	108${}^{{}^{\circ}}C$	0${}^{{}^{\circ}}C$
2	Matrix	22${}^{{}^{\circ}}C$	30 wt. % 80 ${}^{{}^{\circ}}$C	-	103${}^{{}^{\circ}}C$	−5 ${}^{{}^{\circ}}C$
3	Matrix	22${}^{{}^{\circ}}C$	30 wt. % 80 ${}^{{}^{\circ}}$C	10 wt. % hBN	86${}^{{}^{\circ}}C$	−22 ${}^{{}^{\circ}}C$
4	Dam and Fill	22${}^{{}^{\circ}}C$	90 wt. % 80 ${}^{{}^{\circ}}$C	10 wt. % hBN	103${}^{{}^{\circ}}C$	−5 ${}^{{}^{\circ}}C$

## Appendix A

**Table A1:** Results from MATLAB script simulation of each combination of the selected sugar alcohols sorted by their melting point.

A	B	Mol. Fract. A	$T_{m,eut}$ ${}^{{}^{\circ}}$C	$\Delta h_{fus,eut}$ J g${}^{-1}$	Mol. Mass g mol${}^{-1}$	Density g cm${}^{-3}$	Δ$h_{fus,eut}$ J cm${}^{-3}$
Isomaltol	Pentaerythritol	0.345	68	108,299	207,907	1.497	77,997
Pentaerythritol	Xylitol	0.538	74	203.562	143.540	1.453	206.097
Sorbitol	Pentaerythritol	0.457	75	181.569	157.202	1.444	166.733
Isomaltol	Xylitol	0.386	75	318.695	226.328	1.586	223.273
Sorbitol	Xylitol	0.493	76	359.779	166.944	1.510	325.449
Pentaerythritol	Ribitol	0.550	78	191.598	143.347	1.456	194.646
Lactitol-Monohydr.	Pentaerythritol	0.425	78	277.390	232.197	1.521	181.685
Xylitol	Ribitol	0.543	78	373.817	152.150	1.525	374.570
Lactitol-Monohydr.	Xylitol	0.425	78	468.105	241.450	1.592	308.689
Isomaltol	Sorbitol	0.381	79	291.766	243.903	1.572	188.089
Isomaltol	Ribitol	0.398	79	306.057	228.661	1.594	213.314
Sorbitol	Ribitol	0.525	79	349.925	167.914	1.514	315.562
Arabinitol	Xylitol	0.413	80	400.135	152.150	1.522	400.284
Lactitol-Monohydr.	Sorbitol	0.442	80	446.612	261.856	1.584	270.168
Lactitol-Monohydr.	Ribitol	0.477	80	469.447	252.426	1.606	298.737
Sorbitol	Arabinitol	0.569	81	375.376	169.231	1.511	335.109
Arabinitol	Pentaerythritol	0.439	82	215.240	143.169	1.453	218.382
Isomaltol	Arabinitol	0.422	82	338.373	233.181	1.595	231.392
Lactitol-Monohydr.	Arabinitol	0.519	82	508.401	261.199	1.611	313.491
Arabinitol	Ribitol	0.456	83	392.392	152.150	1.528	393.997
Erythritol	Xylitol	0.295	85	391.739	143.277	1.499	409.935
Isomaltol	Lactitol-Monohydr.	0.393	85	440.703	355.248	1.690	209.653
Lactitol-Monohydr.	Erythritol	0.631	86	522.224	273.697	1.601	305.561
Sorbitol	Erythritol	0.665	87	363.171	162.083	1.483	332.351
Lactitol-Monohydr.	Maltol	0.671	87	455.519	284.504	1.667	266.893
Maltol	Xylitol	0.227	88	338.919	146.244	1.543	357.514
Erythritol	Ribitol	0.354	88	382.239	141.530	1.502	405.576
Isomaltol	Erythritol	0.478	89	321.040	228.308	1.565	220.024
Sorbitol	Maltol	0.737	90	298.911	167.399	1.532	273.489
Arabinitol	Erythritol	0.633	90	422.052	141.129	1.497	447.825
Maltol	Ribitol	0.277	92	316.570	144.931	1.555	339.646
Erythritol	Pentaerythritol	0.405	93	198.381	130.461	1.418	215.608
Isomaltol	Maltol	0.499	93	205.255	234.954	1.655	144.573
Mannitol	Xylitol	0.037	94	389.156	153.251	1.520	385:980
Lactitol-Monohydr.	Mannitol	0.956	94	584.582	354.371	1.682	277.549
Arabinitol	Maltol	0.718	95	360.194	144.813	1.552	385.973
Lactitol-Monohydr.	Myo-Inositol	0.979	95	580.763	358.551	1.697	274.910
Lactitol-Monohydr.	Dulcitol	0.985	95	585.714	359.598	1.687	274.724
Sorbitol	Mannitol	0.947	98	349.826	182.170	1.501	288.251
Sorbitol	Myo-Inositol	0.978	99	338.200	182.125	1.512	280.779
Sorbitol	Dulcitol	0.982	99	342.700	182.170	1.499	282.077
Mannitol	Ribitol	0.047	100	374.419	153.573	1.530	372.908
Myo-Inositol	Xylitol	−0.156	100	380.615	147.794	1.439	370.661
Myo-Inositol	Ribitol	0.017	101	364.648	152.634	1.539	367.622
Arabinitol	Mannitol	0.937	101	435.779	154.054	1.525	431.292
Arabinitol	Myo-Inositol	0.974	102	425.090	152.876	1.538	427.749
Arabinitol	Dulcitol	0.977	102	430.973	152.829	1.524	429.695
Dulcitol	Xylitol	−0.300	103	302.990	143.149	1.535	324.897
Dulcitol	Ribitol	−0.046	103	351.251	150.766	1.533	357.100
Maltol	Pentaerythritol	0.387	106	105.438	132.263	1.483	118.200
Erythritol	Maltol	0.644	107	335.891	123.540	1.511	410.691
Erythritol	Mannitol	0.868	115	437.013	130.073	1.459	490.278
Erythritol	Myo-Inositol	0.952	117	414.003	124.909	1.478	489.974
Erythritol	Dulcitol	0.946	117	427.683	125.379	1.451	494.982
Isomaltol	Mannitol	0.761	123	304.625	305.552	1.649	164.436
Isomaltol	Dulcitol	0.854	132	280.354	320.692	1.658	144.941
Isomaltol	Myo-Inositol	0.871	133	240.361	323.128	1.735	129.062
Mannitol	Pentaerythritol	0.292	134	205.095	149.590	1.432	196.364
Maltol	Mannitol	0.687	138	313.645	143.653	1.589	346.870
Maltol	Myo-Inositol	0.831	148	225.757	135.228	1.691	282.252
Maltol	Dulcitol	0.821	148	273.372	136.138	1.593	319.917
Dulcitol	Pentaerythritol	0.249	151	198.540	147.608	1.414	190.246
Mannitol	Dulcitol	0.696	156	599.643	182.170	1.505	495.338
Mannitol	Myo-Inositol	0.829	161	547.367	181.826	1.609	484.329
Myo-Inositol	Pentaerythritol	0.238	164	131.276	146.617	1.549	138.685
Dulcitol	Myo-Inositol	0.711	178	571.307	181.588	1.635	514.280
